# Aberrant right coronary artery in a grown up congenital cardiac patient, successfully treated 46 years earlier with a double Starr-Edwards silastic ball valve replacement: a case report

**DOI:** 10.1186/s12872-020-01351-1

**Published:** 2020-01-29

**Authors:** Andrea Ponsiglione, Gianrico Spagnuolo, Gabriella Spagnuolo, Arnaldo Stanzione, Carmela Nappi, Serena Dell’Aversana, Antonio Spagnuolo, Alberto Cuocolo, Massimo Imbriaco

**Affiliations:** 1 Department of Advanced Biomedical Sciences, University of Naples “Federico II”, Via Pansini 5, 80131 Naples, Italy; 2 Department of Neurosciences, Reproductive and Odontostomatological Sciences, University of Naples “Federico II”, Naples, Italy

**Keywords:** Aortic valve replacement, Mitral valve replacement, Congenital heart disease, Coronary anomaly, Case report

## Abstract

**Background:**

The Starr-Edwards ball valve prosthesis was successfully introduced in 1961–62 and largely used for aortic and mitral valve replacement. Even if Starr-Edwards valves have been widely replaced in clinical practice by other mechanical valves, they define a standard concerning long-term durability.

**Case presentation:**

We describe the case of a 55-year-old man referred to our Department to perform a cardiac computed tomography (CCT), to better evaluate a severe dilation of ascending aorta discovered at echocardiography. The patient had been surgically treated 46 years earlier to correct a supra-cristal type ventricular septal defect. Both mitral and aortic valves were replaced, respectively due to bacterial mitral endocarditis and a fibrous sub-valvular aortic stenosis. In addition, the right coronary artery (RCA) was found to arise from the left coronary sinus.

**Conclusion:**

We report the longest lasting durability (46 years) of aortic and mitral Starr-Edwards valves successfully implanted in a patient simultaneously carrying a malignant anomalous origin of RCA.

## Background

The Starr-Edwards silastic ball valve prosthesis has been widely used for aortic and mitral valve replacement since its introduction in the 1960s [1, 2]. Since then, more than 200,000 Starr-Edwards silastic ball valves have been implanted worldwide [3, 4]. McGoon et al. reported that these prostheses implants were durable over a prolonged follow-up period, while thromboembolism remained a persistent problem [5]. Different kind of complications have been reported including haemolytic anaemia, valve dysfunction caused by granulomatous hyperplasia, degeneration or lipid infiltration of the silicon ball and wearing out of the cloth cover [6, 7].

We hereby describe the longest lasting durability (46 years) of aortic and mitral Starr-Edwards valves successfully implanted in a patient simultaneously carrying a malignant anomalous origin of RCA, incidentally discovered at surgery. This unique association has never been reported in the literature.

## Case presentation

The patient is a 55-year-old man who underwent a previous surgery to correct a supra-cristal type ventricular septal defect 46 years earlier. During the surgical procedure, mitral valve resulted to be markedly involved in a destructive process, with ruptured chordae, as consequence of a bacterial endocarditis. Therefore, a size 2 silastic Starr-Edwards valve was implanted. Moreover, a localized fibrous subvalvular aortic stenosis, due to a ring was identified. The ring was excised, and a size 8 Starr-Edwards valve was implanted. In addition, RCA was found to arise from the left coronary sinus at the commissural attachment of the cusp. The patient was subsequently discharged with anticoagulant therapy with phenprocoumon, considering a target International Normalized Ratio of 4.0, without need of any surgical correction of the anomalously originating RCA from left coronary sinus.

**Fig. 1:**
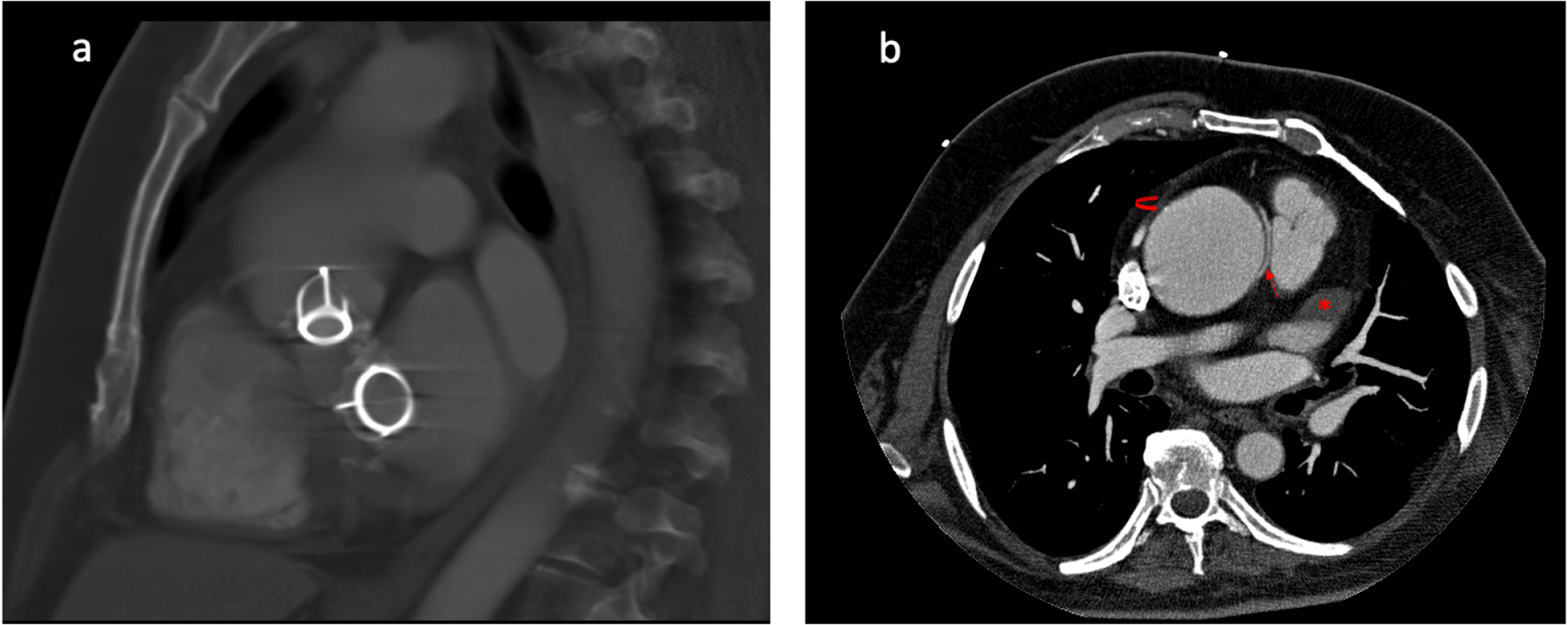
Cardiac Computed Tomography. Sagittal reconstruction showing both mitral and aortic silastic Starr-Edwards valves (1**a**); axial view showing ascending aortic dilation (curved arrow), the anomalous origin of the right coronary artery (straight arrow) coursing between the aortic root and the main pulmonary artery and a thrombus in the left atrial appendage (asterisk) (1**b**)

Clinical follow-up was unremarkable, until the age of 55, when the patient underwent an echocardiography showing a severe dilation of ascending aorta. No trans-valvular leakage in the aortic position or mitral valvular regurgitation was identified; in addition, no thrombotic material was detectable at the prostheses site. Thus, the patient was scheduled for a cardiac computed tomography (CCT) for further diagnostic evaluation. CCT was performed using a 64 slices scanner (Aquilion, Toshiba) and allowed to appreciate both mitral and aortic silastic Starr-Edwards valve in correct position, confirming a significant dilation of the ascending aorta with a maximum transverse diameter of 6.2 cm (Fig. 1a-b). The anomalous origin of RCA from left coronary sinus, 1 cm above the origin of the left coronary artery and its course between the aortic root and main pulmonary artery were clearly identified (malignant variant) (Fig. 1a-b). A thrombus in the left atrial appendage was also detected (Fig. 1a-b). This patient has been living for 46 years with the Starr-Edwards mitral and aortic valves, in association with a rare coronary abnormality. Furthermore, the patient never showed any sign of prosthetic dysfunction, embolism, infection, periprosthetic leaks or hemolysis. Of note, the patient did not show any cardiac symptoms neither related to the right coronary abnormality nor to the ascending aortic aneurysm (AAA). Although medical treatments can slow the enlargement of AAA, the mainstay of prevention of aortic dissection is surgical repair when the aortic diameter expands to 5.5 cm or more [8]. In our case, the patient is still asymptomatic and refuses surgical correction of the AAA and is strictly monitored with CCT every 6 months; the patient is under medical treatment with anticoagulation therapy and anti-hypertensive drugs for mild hypertension.

## Discussion and conclusions

The Starr-Edwards ball valve prosthesis was successfully introduced in 1961–62 and largely used for aortic and mitral valve replacement [4, 5]. It is one of the artificial valves with very long-term results [9]. Godje et al. reported a 30-year survival rate respectively of 19.9% in patients with aortic valve replacement and 22.6% in patients with mitral valve replacement [3]. Thromboembolism is a major factor contributing to the overall mortality and morbidity of patients who underwent valve replacement. In this setting, the necessity of long-term anticoagulation is controversial. Godje et al. found, in their patient’s population, that only 65.7% of patients with Starr-Edwards aortic valves received anticoagulation therapy, whereas patients with mitral valve replacement were treated with anticoagulant therapy in 90.7% of cases [3]. McGoon et al. found in a cohort of 336 patients that an adequate status of anticoagulation significantly reduced the risk of systemic embolism only in patients with a mitral valve prosthesis, suggesting a difference in the pathogenesis of emboli originating from mitral and aortic valve prostheses [5]. Nowadays, all surviving patients are treated with oral anticoagulation. Even if Starr-Edwards valves have been widely replaced in clinical practice by other mechanical valves, they define a standard concerning long-term durability. Abad et al. reported a case of a patient who lived almost 50 years after aortic valve replacement with a Starr-Edwards Caged-Ball Valve [10]. Yalcinkaya et al. described a still functioning Starr-Edwards mitral valve implanted 41 years before [11]. Starr et al. described that their longest-lived survivor had an original ball-valve prosthesis in the aortic position for 51.7 years, whereas the longest-functioning mitral valve lasted for 44.4 years [12]. Our patient has been living for 46 years with both Starr-Edwards mitral and aortic valves, without showing any sign of prosthetic dysfunction, embolism, infection, periprosthetic leaks or hemolysis.

The case presented is unique since our patient also shows a RCA anomalously originating from the left coronary sinus and coursing between the aortic root and the main pulmonary artery (malignant variant). Congenital anomalies of the coronary arteries are relatively uncommon, occurring in 0.2 to 1.2% of the population. These anatomic variants could be asymptomatic, but sometimes could represent an important cause of chest pain, determining hemodynamically significant abnormalities, until in worst cases sudden cardiac death. The anomalous right coronary origin may have inter-arterial (the most common one), retro-aortic, prepulmonic or septal (subpulmonic) course. The incidence of this variant is unknown. In particular, Yamanaka et al. reported an incidence of 0.1% in their population, who underwent coronary angiography [13]. Erol et al. reported a prevalence rate for RCA branching from the left coronary sinus of 0.43% in patients undergoing CCT [14]. The incidence of clinical events related to RCA abnormal origin, such as angina pectoris, myocardial infarction, or sudden cardiac death, is not known either and it is generally estimated from necropsy patients [15, 16]. Several explanations have been proposed for the association of this anatomic variant and clinical events including acute angulation at the origin, compression of the vessel between the aorta and pulmonary artery, slit like ostium and intramural proximal intussusception of the ectopic artery at the aortic root [17]. Nevertheless, the choice between surgical treatment or a conservative strategy is controversial [18–20]. Proposed treatment options include revascularization, translocation of the RCA to the aorta, ostioplasty and bypass grafting [21]. Since our patient did not show any cardiac symptoms a conservative strategy was adopted.

In conclusion, we reported the longest lasting durability (46 years) described in the English literature of a double mitral-aortic Starr-Edwards silastic ball valve in association with an anomalous malignant origin of the RCA, coursing between the aortic root and the main pulmonary artery.

## Data Availability

The datasets used and/or analyzed during the current study are available from the corresponding author on reasonable request.

## Abbreviations

AAA: Ascending aortic aneurysm
CCT: Cardiac computed tomography
RCA: Right coronary artery
